# Optimization of Conditions for Expression of Dengue Serotype 2 EDIII Protein in *Escherichia coli* and Immune Responses of Adjuvant-Free EDIII Ferritin Nanoparticles Against Dengue Virus in BALB/c Mice

**DOI:** 10.3390/v17010129

**Published:** 2025-01-17

**Authors:** M.S.B.W.T.M. Nipuna Sudaraka Tennakoon, Kyoung-Ho Lee, Hye-Mi Lee, Jae-Yeon Park, Hyun-Jin Shin

**Affiliations:** 1 Laboratory of Infectious Diseases, College of Veterinary Medicine, Chungnam National University, Daejeon 34134, Republic of Korea; nipunatennakoon55@gmail.com (M.N.S.T.); hanulvitt@naver.com (K.-H.L.); hyemi0728@gmail.com (H.-M.L.); wodus5818@naver.com (J.-Y.P.); 2 CellEnVax. Co., Ltd., Daejeon 34134, Republic of Korea

**Keywords:** dengue, ferritin nanoparticles, EDIII, *Escherichia coli* expression, protein aggregates, immune responses

## Abstract

Self-assembling ferritin nanoparticle technology is a widely used vaccine development platform for enhancing the efficacy of subunit vaccines by displaying multiple antigens on nanocages. The dengue virus (DENV) envelope domain III (EDIII) protein, the most promising antigen for DENV, has been applied in vaccine development, and it is essential to evaluate the relative immunogenicity of the EDIII protein and EDIII-conjugated ferritin to show the efficiency of the ferritin delivery system compared with EDIII. In this study, we optimized the conditions for the expression of the EDIII protein in *E. coli*, protein purification, and refolding, and these optimization techniques were applied for the purification of EDIII ferritin nanoparticles. Thus, purified DENV2 EDIII and EDIII human ferritin heavy chain nanoparticles were immunized intramuscularly into BALB/c mice without an adjuvant, and the immunogenicity was analyzed using IgG ELISA and a serum-neutralizing assay. Purified, properly refolded, aggregate-free EDIII and EDIII ferritin proteins were obtained, and ferritin nanoparticles were identified using an electron microscope. By analyzing the immunogenicity of mouse serum, EDIII ferritin generated significantly higher IgG responses and neutralizing activity than EDIII-immunized mice. The IgG ELISA results confirmed that EDIII ferritin can induce a significantly higher IgG titer (O.D.:1.8) than EDIII (O.D.:0.05). Furthermore, EDIII ferritin produced a neutralizing titer of 1:68, whereas EDIII protein produced an average titer of 1:16, which is the serum dilution that inhibited 90% of the viruses. The longevity of the immune responses was analyzed using the serum obtained 2 months after the final immunization, and the results confirmed that EDIII ferritin induced constant immunity throughout the period.

## 1. Introduction

Dengue virus (DENV) is transmitted mainly through the female mosquitos of Aedes species, and infected mosquitos can transfer the DENV to a non-infected human from the salivary gland of mosquitos to the host cells through the mosquito bite [1]. DENV is categorized into the genus *Flavivirus* and the family *Flaviviridae* [2]. DENV is classified into four antigenically distinct serotypes as follows: DENV-1, DENV-2, DENV-3, and DENV-4. These serotypes are further categorized based on their surface antigens and can provide specific immunity [2]. Dengue virus (DENV) has a 10–11 kb length single-stranded positive-sense RNA genome, including the 5′ end cap, and it encodes ten proteins [3]. The viral genome encodes three structural proteins (Envelope, Capsid, and Membrane) and seven non-structural proteins (NS1, NS2A, NS2B, NS3, NS4A, NS4B, and NS5) [3]. DENV has a long history of outbreaks and epidemics. The first recorded dengue-like epidemics occurred in the late 18th century in Asia, Africa, and North America [4]. According to World Health Organization (WHO) reports, in 2023, there were over five million cases worldwide, including 80 countries, signifying its impact on global health. DENV causes a wide spectrum of illnesses in humans, ranging from asymptomatic infections to life-threatening conditions. The most common manifestations include dengue fever (DF), a self-limiting febrile illness, and more severe forms, such as dengue hemorrhagic fever and dengue shock syndrome (DSS) [3].

Although prevention and control are the major disease control measures, vaccines are important for reducing disease severity. The WHO has licensed two vaccines, Dengvaxia and Qdenga, and several other vaccines are currently being evaluated. According to previous studies, the vaccine efficacy and safety of Dengvaxia and Qdenga were reported to vary according to the target immunized population, and serotype-specific immunity was also low with some dengue serotypes [5]. Therefore, the development of new vaccine strategies with outstanding efficacy and safety is crucial to confront the increasing disease occurrence and future vaccine requirements. According to previous reports, inactivated, live-attenuated nucleic acid, viral vectors, virus-like particles, and subunit vaccines have been developed [6]. Compared with other vaccine types, subunit vaccines have advantages such as a lack of potential for reverting to the virulent form and no extreme temperature requirement for storage, although low immunogenicity is a major concern. Therefore, the administration of protein subunits with adjuvants and immunomodulatory delivery systems, such as nanoparticle vaccines, has been shown to elicit significant immune responses [7]. The polymeric representation of antigens on the surface of the nanoparticle allows tight and prolonged binding with B cell receptors compared with the single antigen following significant B- and T-cell responses [8]. Among nano-vaccine carriers, self-assembling ferritin nanoparticle technology is an emerging nano-platform that has been used in multiple studies, and robust immune responses have been elucidated [9,10,11,12,13]. Therefore, developing vaccine strategies that integrate ferritin and dengue antigenic molecules can have a fruitful impact on the future development of dengue vaccines.

**Figure 1:**
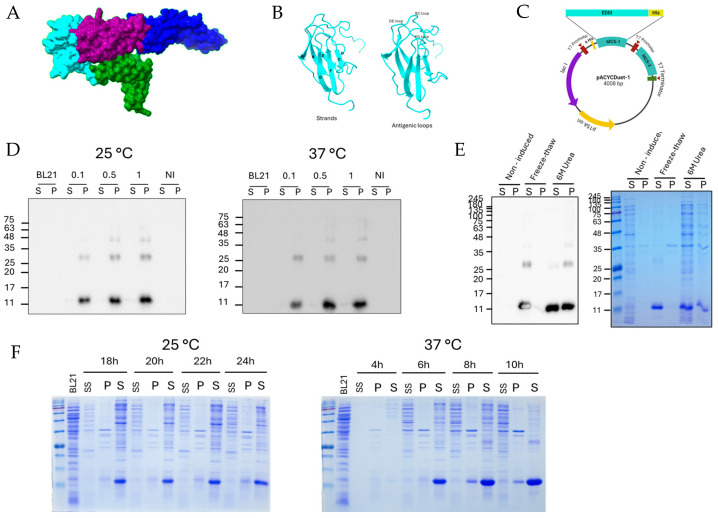
Dengue EDIII cloning and expression in *E. coli*. (**A**) Envelope protein domains (Purple-Domain I, Blue-Domain II, Cyan-Domain III, and Green-Stem domain). (**B**) Envelope domain III strands and antigenic loops. (**C**) The EDIIII gene was cloned into MCS1 of the pACYCDuet1 vector. (**D**) The cloned plasmid was transformed into *E. coli* BL21 strain, the expression of EDIII protein was analyzed under different IPTG concentrations (1 mM, 0.5 mM, and 0.1 mM) in LB medium, and samples prepared in mild buffer were analyzed using Western blotting. (**E**) Insoluble EDIII protein expressed as inclusion bodies were solubilized using two different methods: freeze–thaw and 6 M urea buffer, followed by an analysis of the solubility of EDIII using Western blotting and SDS-PAGE. (**F**) The EDIII protein was expressed in autoinduction media at different temperatures and times following analysis of the harvested samples by SDS-PAGE. (S: supernatant, P: pellet, SS: sample sonicated supernatant with PBS, NI: Non-induced).

The envelope protein is the major protein of the dengue virus and contains neutralizing epitopes and is divided into four major regions: envelope domain I (EDI), envelope domain II (EDII), envelope domain III (EDIII), and transmembrane domain (Figure 1A) [3]. Although envelope proteins are strongly targeted antigenic elements by antibodies produced in infected patients, and the dengue virus envelope protein is the primary antigenic molecule used in subunit vaccine design, the risk of antibody-dependent enhancement (ADE) can be reduced using the EDIII protein because it does not contain non-neutralizing and cross-reactive epitopes [14]. The assembly of the EDIII protein generates seven major protein strands with three major antigenic loops: BC, DE, and FG (Figure 1B). Previous research groups have identified different major antigenic epitopes on the EDIII protein [15,16,17,18]. Therefore, the EDIII protein has been used in different vaccine studies as a primary antigenic molecule [19,20,21]. Furthermore, it has been reported that the immunogenicity of subunit vaccines can be increased significantly using the nanoparticle vaccine platform [8,9,10,11,12,13,14,15,16,17,18,19,20,21,22], and ferritin can be used to enhance the immunogenicity of EDIII by displaying multiple EDIII molecules on the ferritin cage. However, limited data are available on the expression, purification, and immunogenicity of EDIII and EDIII ferritin nanoparticles. Therefore, the primary objective of this study was to optimize *E. coli* expression, purification, and refolding of DENV2 EDIII and EDIII ferritin nanoparticles and to elucidate the immune responses of DENV2 EDIII ferritin nanoparticles compared to those of DENV2 EDIII.

Here, we report new buffer conditions that can be used to purify DENV EDIII proteins to eliminate protein aggregates, which can be used to optimize the purification of other viral proteins for the initial confirmation of subunit vaccine immunity. The robust immunostimulatory activities of DENV2 EDIII ferritin nanoparticles compared to DENV EDIII provide greater insight into the development of ferritin nanoparticle vaccines targeting all serotypes and the application of this method in vaccine development for other *Flaviviruses*.

## 2. Materials and Methods

### 2.1. Cell Culture, Virus, and Bacterial Strains

Vero and HELA cells were cultured in Minimum Essential Medium (MEM) (Welgene, Gyeongsan-si, Republic of Korea) supplemented with 10% fetal bovine serum (FBS), 1% antibiotic-antimycotic (AA), and 1% HEPES. The *E. coli* strain BL21 was used for the overexpression of recombinant protein. The DENV-2/KBPV-VR-29 strain was propagated in Vero cells, as previously reported [9].

### 2.2. Plasmid Construction for DENV2 EDIII and EDIII Ferritin

EDIII and EDIII ferritin genes were cloned into the first multi-cloning site (MCS1) of the prokaryotic expression vector, pACYCDuet1. The gene encoding DENV2 EDIII protein (aa 289–398) was cloned downstream of the T7 promoter and 6 histidine (His) tag of the vector. The gene was amplified with an additional 6 histidine tag in the C-terminal using the forward primer 5′ CGGGATCCGATGGATAAGCTGCAGCT 3′ and reverse primer 5′ AAGCTTGTCGACCTAGTGGTGATGATGGTGATGTTGTCCGATGCTGCTACC 3′, followed by cloning between the *BamH*I and *Sal*I enzyme sites. Two-step cloning was performed to construct an EDIII ferritin plasmid. The first EDIII protein was amplified using the same forward primer mentioned above and reverse primer, including the SSG linker and *EcoR*I enzyme site downstream 5′ AAGCTTGTCGACGAATTCGCCGCTGCTTTGTCCGATGCTGCTACCTT 3′ and was cloned between the enzyme sites *BamH*I and *Sal*I, followed by obtaining the EDIII SSG. Then, two types of human ferritin heavy chain genes were amplified (without the N-terminal His tag and with the N-terminal His tag) using the forward primer 5′ CCGGAATTCACGACCGCGTCCACCTCG 3′ and two reverse primers: 5′ CTTAAGGCGGCCGCTTAGCTTTCATTATCACTGTC 3′ and 5′ CTTAAGCATTATGCGGCCGCTTAGTGGTGATGATGGTGATGGCTTTCATTATCACTGTC 3′, respectively. Subsequently, the amplified genes were inserted into the vector using *EcoR*I and *Not*I enzyme sites to obtain EDIII ferritin and EDIII ferritin 6His.

### 2.3. Protein Expression and Purification

#### 2.3.1. EDIII Protein Expression in *E. coli*

Recombinant plasmids expressing the EDIII protein were transformed into BL21 competent *E. coli* using the heat shock method, after which the cells were spread on an LB plate in the presence of 30 µg/mL of chloramphenicol and allowed to form colonies at 37 °C overnight. A colony was selected and cultured in 10 mL of LB medium containing the same concentration of chloramphenicol for 10–12 h. LB media with antibiotics were divided into 14 mL round bottom tubes (2 mL each). To compare the EDIII protein expression levels in the pellet and soluble fractions, each tube was inoculated with 20 µL (1:100 ratio) of the above cultured *E. coli* and incubated at 37 °C until it reached the optimum (O.D.) value (0.5–0.6). Then, the samples were induced with different IPTG concentrations (0.1, 0.5, and 1.0 mM), and they were cultured at two different temperatures: 37 °C and 25 °C for 5 h and 22 h, respectively. Extraneously to the EDIII samples, the cell-only samples were cultured as negative controls at two different temperatures. All samples were harvested by centrifuging at 5000× *g* for 10 min at 4 °C. The cells were resuspended in a one-tenth volume (500 µL) of mild buffer containing 50 mM NaCl and 20 mM Tris-HCl (pH 7.8), followed by sonication at 50 °C for 5 min using the standard tip Ɵ2 of the ultrasonic homogenizer (SCIENTZ, KUS-650 KBT, Zhejiang, China). The supernatant and pellet fractions were separated by centrifuging at 19,000× *g* for 10 min, and the pellet fraction was suspended in the same buffer as the supernatant for comparison of the expression levels. Expression was analyzed by 12% Western blotting.

#### 2.3.2. Solubilization of Inclusion Bodies

Since it was not essential to induce IPTG, an autoinduction medium was used as the protein expression medium for the following experiments. To solubilize the inclusion body pellet, two methods were used: 6 M urea buffer and 2 M urea freezing–thawing. The 6 M urea buffer was prepared using 6 M urea, 20 mM Tris-HCl, and 0.5 M NaCl at a pH of 7.6. The cell pellet was resuspended in 500 µL of Harsh buffer and sonicated at 50 W for 5 min using the standard tip Ɵ2. The cell suspension was centrifuged at 25,000× *g* for 10 min to recover the supernatant, and the pellet was resuspended in an equal volume (500 µL) of PBS.

In the 2 M urea freezing–thawing method, cell pellets were suspended with 500 µL of PBS, and it was sonicated at the power of 50 W for 5 min using standard tip Ɵ2. After centrifugation at 20,000× *g* to pellet the inclusion bodies, they were suspended in 300 µL of 1 M urea wash buffer and then centrifuged at 20,000× *g* for 5 min to recover the pellet. This washing step was repeated 3 times, and finally, the pellet was obtained. The pellet was subsequently suspended in 200 µL of 2 M urea sonication buffer and frozen at −20 °C overnight. The frozen suspension was thawed at room temperature and centrifuged at 20,000× *g* for 10 min to recover the supernatant containing solubilized proteins and the pellet containing inclusion bodies. The protein solubilities in 2 M urea and 6 M urea buffer were evaluated using SDS-PAGE and Western blotting.

#### 2.3.3. Evaluation of the Optimum Incubation Time for EDIII Expression in Autoinduction Media

The same *E. coli* strain (BL21) expressing the EDIII protein was inoculated into 10 mL of LB chloramphenicol medium and incubated overnight at 37 °C in a shaking incubator. Subsequently, 1 mL of the same LB medium was aliquoted into 14 mL round bottom tubes, and 10 µL (1:100 diluted) of overnight culture was added to each tube. One set of samples was incubated at 25 °C and harvested by centrifugation at 5000× *g* for 5 min at 18, 20, 22, and 24 h after inoculation. Another set was incubated at 37 °C, and the samples were harvested under the same conditions at 4, 6, 8, and 10 h post-inoculation. The samples were prepared according to the 2 M freezing–thawing method, and the expression levels of EDIII in PBS-sonicated supernatant, freezing–thawing pellet, and supernatant were analyzed using SDS-PAGE.

#### 2.3.4. Purification of EDIII Protein

*E. coli* expressing the EDIII protein were pellet from a 50 mL culture. A solubilized EDIII protein supernatant was obtained using the previously mentioned 2 M urea freezing–thawing method; imidazole (Sigma-Aldrich, 15513) was added to the supernatant to a final concentration of 10 mM, and the pH was adjusted to 7.6, followed by filtration using a 0.2 µm filter.

Proteins were purified using immobilized metal affinity chromatography (IMAC). His affinity columns (Thermo Fisher Scientific, 29922, Waltham, MA, USA) were loaded with 1.5 mL of His-tag purification resin (Sigma-Aldrich, 5893682001, Seoul, Republic of Korea) and washed with 14 mL of binding buffer (20 mM Tris-HCl, 0.5 M NaCl, pH 7.5). The filtered sample was loaded into the column and allowed to flow through the resin at a speed of 1 mL/1 min. Washing was performed using 20 mL of washing buffer (20 mM Tris-HCl, 0.5 M NaCl, 20 mM imidazole, pH 7.5). The His-tagged proteins were eluted using 15–20 mL of elution buffer (20 mM Tris-HCl, 0.5 M NaCl, 200 mM imidazole, pH 7.5), and the eluted proteins were filtered through a 0.2 µm filter. The purified proteins were concentrated using Amicon ultracentrifugation filter units. To reduce impurities, two filtrations were conducted. First, the sample was filtered through a 30,000 Da filter, and the filtrate was concentrated using a 10,000 Da filter to obtain a 0.5–1 mL protein supernatant. Protein aggregation was assessed by storage at 4 °C.

#### 2.3.5. Elimination of Protein Aggregates

To prevent protein aggregation, the eluted proteins were dialyzed against refolding buffer (buffer 1) containing 50 mM NaCl, 400 mM L-arginine, 5% glycerol, and 20 mM HEPES (pH 6.4). Before adjusting the pI value of the refolding buffer, the pH was maintained at least 1 unit away from the calculated pI value for a specific protein. The pI value was calculated using an online tool (https://web.expasy.org/compute_pi/, accessed on 15 August 2024). For protein renaturation, a similar volume of the refolding buffer with the elute (1:1) was added dropwise to the protein elute and stored at 4 °C overnight. The properly folded proteins were separated from the aggregated proteins or debris by centrifuging at 19,000× *g* for 15 min. The supernatant was transferred into a pre-wet 10,000 Da dialysis bag (Thermo Scientific, Waltham, MA, USA, 68100) and dialyzed twice against 500 mL of refolding buffer for 8 h by changing the buffer at 4 h intervals. To further eliminate aggregated or dimerized proteins, dialyzed protein was added to 30,000 Da Amicon filter units. Assuming the molecular weight of the EDIII protein (14 kDa), Monomeric EDIII proteins were concentrated using 10,000 Da Amicon filter units. The protein concentration was analyzed using a Bicinchoninic acid (BCA) protein assay kit (iNtRON Biotechnology, #21071, Seongnam-si, Republic of Korea).

#### 2.3.6. Cell Toxicity Analysis and Reduction of Buffer Toxicity

Ten times dilution of the relevant protein with the above buffers was sufficient to prepare at least 200 µg/µL of the protein for immunization. Therefore, as the initial dilution for the cell toxicity assay, the buffers were diluted ten times. The HeLa cells were prepared in 96-well cell culture plates (2 × 10^4^ cells/well) before testing. When the cells achieved 80–90% confluence, serially diluted protein buffers in MEM containing 2% FBS and 1% AA (100 µL/well) were added. After 48 h, the medium was replaced with 100 µL of PBS, and the cells were washed. EZ-Cytox reagent (DoGenBio, EZ-1000) diluted in serum-free MEM (1:9) was added to each well (110 µL/well) and incubated in a CO_2_ incubator at 37 °C for 1 h. The medium was harvested by centrifuging at 12,000 rpm for 3 min, and 100 µL of the harvested media was added to each well of a 96-well cell culture plate, and readings were taken using a plate reader at 450 nm.

To reduce the effect of refolding buffer toxicity on the cells, the DENV2 EDIII protein was dialyzed in a modified refolding buffer (buffer 2) containing 50 mM NaCl, 150 mM L-arginine, 3% glycerol, and 10 mM HEPES (pH 6.4, based on the pI value) instead of using buffer 1. However, other steps were followed without any changes.

#### 2.3.7. EDIII Ferritin Expression and Purification

Based on the conditions used for protein expression and purification of EDIII, all conditions were optimized to obtain EDIII ferritin particles. Briefly, EDIII ferritin inclusion bodies were solubilized using a 2 M urea freezing–thawing method. The proteins were purified using affinity purification followed by elution and were then dialyzed in modified refolding buffer (buffer 2) containing 50 mM NaCl, 150 mM L-arginine, 3% glycerol, and 10 mM HEPES (pH 7.4, based on the pI value). Proteins were concentrated using 100,000 Da Amicon filter units, depending on the molecular weight of the polymerized ferritin (>450 kDa) nanoparticles.

### 2.4. Dynamic Light-Scattering (DLS) Assay

DLS analysis was performed to analyze the size distribution pattern of EDIII ferritin nanoparticles. The samples were centrifuged at 19,000× *g* for 10 min, and the supernatant was recovered. The samples were analyzed using a Zetasizer ZetaPALS^®^ (Brookhaven Instruments, Holtsville, NY, USA).

### 2.5. Transmission Electron Microscopy

The EDIIII ferritin protein sample concentration was adjusted to 1 mg/mL, and the sample was sonicated for 3 min at a power of 50 W. The samples were prepared in a copper grid (TED PELLA, Inc., #01700-F, Redding, CA, USA). A pale-colored side of the copper grid was loaded with ten microliters of the sample and incubated for 10 to 15 min, and excess protein was absorbed into Whatman paper and dried well. Five microliters of EM stain, R1000 UA-Zero™ (Agar Scientific, Rotherham, UK), were loaded onto the copper grid, followed by incubating for 2–3 *s* and removing the staining solution using the Whatman papers. The perfectly dried grid was analyzed and imaged using an atomic resolution transmission electron microscope (JEM-ARM200F).

### 2.6. Mouse Immunization

Six-week-old female BALB/c mice were purchased from Samtaco Laboratories (Samtaco, Republic of Korea) and maintained according to the guidelines of the Animal Approval Committee of the Chungnam National University (CNU), Korea. Five mice were used for each immunization group. Each group of mice was immunized intramuscularly with purified EDIII, EDIII ferritin, or PBS with buffer 2 in a molar-equivalent manner without using an adjuvant. Purified proteins were prepared in PBS, and the final immunizing volume was 100 µL. The EDIII group received 16.4 µg of protein, whereas the EDIII ferritin group received 40 µg. For the PBS group, PBS was mixed with buffer 2, a volume similar to the highest volume used for EDIII and EDIII ferritin administration, and administered to mice in the control group. Considering the first administration day as day 0, the second and third administrations were performed on days 14 and 28, respectively. For immunogenicity analysis, blood was collected one week after the second immunization, one week after the third immunization, and one and two months after the third immunization.

### 2.7. IgG ELISA, SDS-PAGE, Western Blotting

DENV2 EDIII-specific IgG responses were analyzed using the serum obtained at each interval. Purified EDIII proteins (1 µg/µL) were coated on immunoplates, and ELISA was performed, as the mentioned protocol in our previous study [9,23].

SDS-PAGE and Western blotting were performed as described previously [23]. Briefly, supernatant or pellet fractions of the samples were mixed with sample buffer and boiled at 95 °C for 10 min and then loaded into the gel and run for protein separation. For Western blot analysis, the proteins were transferred to a nitrocellulose membrane. To detect His-tagged proteins, rabbit anti- 6his tag primary antibody (Thermo Fisher Scientific, PA1-983B, Waltham, MA, USA) was used, while EDIII proteins were detected with rabbit anti-EDIII primary antibody (Abbexa, abx201289, Cambridge, UK). HRP-conjugated anti-rabbit antibody (CUSABIO, CSB-PA489724, Houston, TX, USA) was used as the secondary antibody. Membranes were analyzed using a chemiluminescence imaging system (Atto, LuminoGraph II, Tokyo, Japan).

To analyze the SDS-polyacrylamide gels, they were stained with a Coomassie staining solution for 2 h, and a destaining solution (distilled water/methanol/acetic acid = 7:2:1) was used to wash the excess stain.

### 2.8. Virus Neutralization Assay

Vero cells were seeded in 96-well plates (1 × 10^4^ cells/well) the day before the test. Serum was incubated at 56 °C for 30 min to inactivate the complement proteins, and the inactivated serum samples were diluted in the dilution medium (MEM, 2% FBS, and 1% AA), preparing a 2-fold dilution series (1:8 to 1:2056). Dengue serotype 2 virus was diluted to 3 × 10^3^ TCID50/mL in a dilution medium. Fifty microliters of the prepared viral and serum samples were mixed and incubated in a CO_2_ incubator at 37 °C for 90 min. Thereafter, the medium in the prepared Vero cell plate was replaced with the neutralized virus, and the plates were incubated in an incubator for 5 days for cytopathic effect (CPE). Four wells were used for each serum dilution. The cells were fixed using 10% formalin solution and stained with 0.05% crystal violet staining solution. The absorbance values were measured for the stained plates, as described previously [24]. Percent virus inhibition of the sample wells was calculated against the negative control wells, and only wells showing ≥ 100% virus inhibition were taken as the surviving wells. Finally, The maximum serum dilution that reduced the CPE by an average of 90% was graphed as the neutralizing titer.

### 2.9. Statistical Analysis

Statistical analyses were performed using GraphPad Prism (GraphPad Software, V8.0, San Diego, CA, USA). A Student’s *t*-test was used to compare the statistical significance of the two groups based on the *p*-values. The statistical significance levels are indicated as *** *p* < 0.001, ** *p* < 0.01, * *p* < 0.05, and ns *p* ≥ 0.05.

## 3. Results

### 3.1. Cloning and Expression of EDIII Protein as an Insoluble Protein

Figure 1A shows the location of the major domains of the envelope protein. The EDIII protein is the major domain and is composed of neutralizing antibody epitopes compared with the other domains and is arranged in the loop regions (Figure 1B). Amino acid residues 289 to 398 represent the EDIII protein of envelope protein, and C-terminal 6 histidine tags were inserted in the MCS1 of the pACYCDuet1 vector, as mentioned in Figure 1C. The expression and solubility of EDIII in mild buffer were evaluated by Western blotting (Figure 1D). At 25 °C and 37 °C, the EDIII protein was expressed in the pellet fraction, and the expression level at 37 °C was higher than that at 25 °C. Furthermore, the EDIII expression level was significant when the IPTG concentration was 0.5 mM (Figure 1D). Compared with the expression of EDIII in the pellet fraction, expression in the soluble fraction was significantly lower, although a smeared band for EDIII was detected in the supernatant fraction after incubation at 37 °C (Figure 1D). The overall evaluation of the Western blot results indicated that EDIII was insoluble in mild buffers at 25 °C and 37 °C.

### 3.2. Solubilization of EDIII Protein Using 6 M Urea and 2 M Urea Freezing–Thawing Methods

Harsh buffers containing 6 M or 2 M urea were applied as chaotropic agents because mild buffer conditions were not sufficient for the solubilization of the inclusion body pellet. According to the Western blot images and SDS-PAGE results in Figure 1E, both the 6 M urea and freezing–thawing methods increased the solubility of the EDIII protein. In the 2 M urea method, the expression of EDIII protein in the soluble fraction was significantly higher than that in the pellet fraction. However, the 6 M urea method did not affect the total solubility of the EDIII protein; a significant fraction of protein was retained in the pellet fraction compared to the freezing–thawing method (Figure 1E). Moreover, considering the SDS-PAGE results, the freezing–thawing method could significantly reduce the impurities in the soluble fraction compared to the 6 M urea method (Figure 1E right).

To obtain the optimum amount of soluble expressed proteins from *E. coli* cultured in autoinduction media, the critical incubation time and temperature were evaluated. Incubation of *E. coli* in autoinduction media at both 25 °C and 37 °C resulted in significant expression of the soluble EDIII protein (Figure 1F). At 25 °C, EDIII expression in the pellet fraction was low. At time points 6 h, 8 h, and 10 h, significant expressions of the EDIII protein were observed in the samples incubated at 37 °C; thus, the highest expression level was reported in the samples incubated for 8 and 10 h (Figure 1F). Comparing the temperatures of 25 °C and 37 °C, incubation at 37 °C for 8–10 h showed a significant level of soluble EDIII proteins.

### 3.3. Protein Purification and Elimination of Protein Aggregates

**Figure 2:**
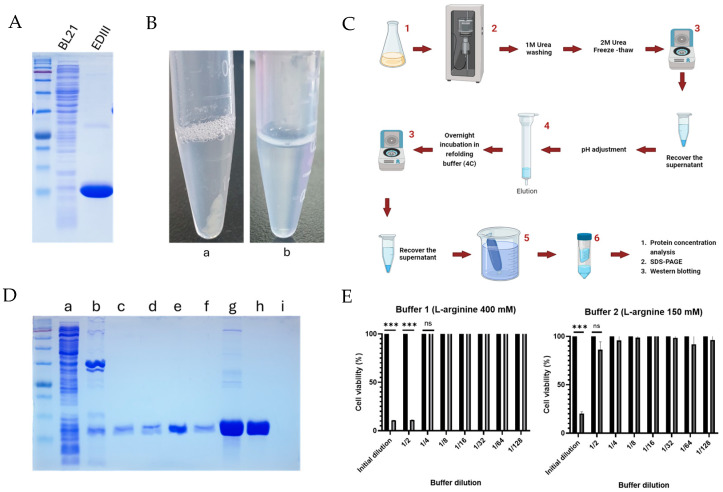
Purification of EDIII protein and elimination of protein aggregates. (**A**) The EDIII protein was purified using His affinity purification, and the purity was analyzed by SDS-PAGE. (**B**) Visibility of insoluble protein aggregates in purified proteins (a) and absence of protein aggregates in properly refolded proteins in L-arginine buffer (b) after storage at 4 °C. (**C**) Schematic representation of protein expression, purification procedures, and dialysis for elimination of protein aggregates. 1—*E. coli* 50 mL culture in autoinduction media; 2—Sonication; 3—centrifugation at 19,000× *g* for 15 min; 4—His affinity purification; 5—dialysis in refolding buffer; 6—concentration of proteins using Amicon centrifugal filter units. (**D**) Samples from different purification steps were analyzed using SDS-PAGE. a—sonicated supernatant; b—pellet after freezing and thawing in 2 M urea; c—supernatant after freezing and thawing in 2 M urea; d—protein after refolding in refolding buffer; e—His affinity purified eluate; f—proteins after dialysis in refolding buffer; g—aggregated or dimerized proteins eliminated by 30 K Amicon filter; h—non-aggregated monomeric EDIII proteins in the elute of 30 K Amicon filter recovered by 10 K Amicon filter; i—10 K Amicon filter elute. (**E**) The toxicity of the refolding buffer (buffer 1) and modified refolding buffer (buffer 2) were evaluated in Hela cells. The statistical significance of the control and buffer samples was evaluated using Student’s *t*-test (*p* < 0.001 = *** and *p* ≥ 0.05 = ns).

The purity of the EDIII protein was analyzed, and monomeric EDIII proteins with the expected molecular weight (14 kDa) and purity were detected (Figure 2A). Although the purity of the protein was significant, storing the protein samples at 4 °C induced the formation of protein aggregates through the misfolding of solubilized proteins (Figure 2B a). These protein aggregates were soluble at different pH values; however, they were regenerated with extensive storage time. Therefore, the purification procedure was modified by using additional refolding and dialysis steps (Figure 2C). Particularly, two steps were important for the proper refolding of proteins while maintaining the other conditions constant. They included the addition of refolding buffer into the protein elution, incubation with refolding buffer after elution, and the final dialysis step with L-arginine and glycerol-containing refolding buffer with an appropriate pH value to remove excess salts. (Figure 2C, step5). At each step of the new protein purification procedure, the expression of the EDIII protein was detected using SDS-PAGE (Figure 2D). As shown in Figure 2D, most of the aggregated or dimeric EDIII proteins were eliminated using 30,000 Da (30 K) Amicon filters, and monomeric EDIII proteins were recovered from filtration through 10,000 Da (10 K) Amicon filter units. Finally, aggregate-free proteins were obtained through refolding and dialysis; however, the cell toxicity analysis of refolding buffer 1 revealed that it could cause significant cell toxicity (Figure 2E, left). Therefore, refolding buffer 2 was prepared using low molar components of all materials, and it reduced cell toxicity significantly compared to refolding buffer 1 (Figure 2E, right). Refolding buffer 2 was used for refolding and dialysis of the EDIII protein, and it showed better protein refolding properties, resulting in aggregate-free proteins stored at 4 °C (Figure 2D b). The concentration of the purified EDIII protein was 1.5 mg/mL, as obtained from the BCA assay.

### 3.4. Construction of Stably Expressed EDIII Ferritin

**Figure 3:**
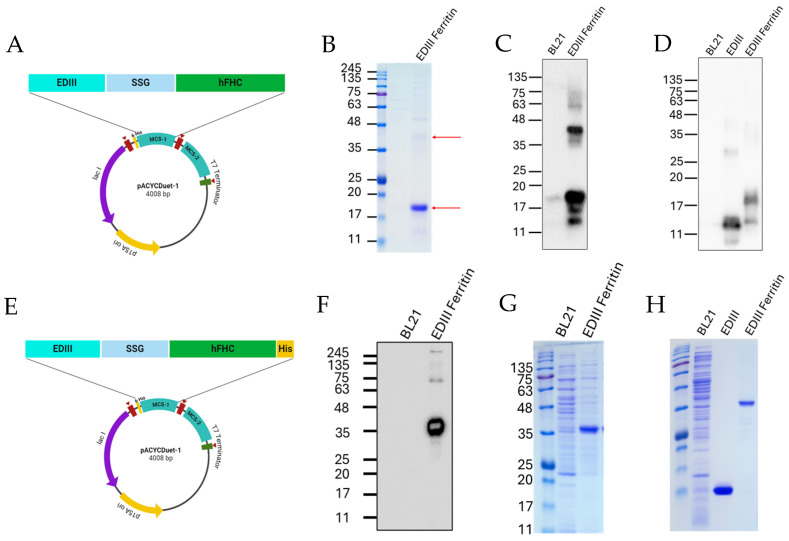
Modification of the EDIII ferritin construct for stable expression. (**A**) EDIII linked to the ferritin gene through the SSG linker was cloned into MCS 1 of the pACYCDuet-1 vector. (**B**) The expression of the EDIII ferritin was analyzed by SDS-PAGE (upper arrow—expected band; lower arrow—resulting major band). (**C**) Blotted EDIII ferritin was detected with an anti-His antibody to identify the cleavage of EDIII and ferritin. (**D**) Blotted EDIII ferritin was detected using an anti-DENV2 EDIII antibody to confirm the cleaved EDIII of the full construct. (**E**) A modified plasmid was generated, including an additional 6-histidine tag in the C-terminal of EDIII ferritin. (**F**) Complete EDIII ferritin protein of the expected size was detected in the Western blot membrane treated with anti-His antibody. (**G**) A complete expression of EDIII ferritin was confirmed by SDS-PAGE. (**H**) The purity of the His-purified EDIII and EDIIII ferritin was confirmed by SDS-PAGE before mouse immunization.

To generate ferritin nanoparticles displaying EDIII proteins, the C-terminus of the EDIII protein was connected to the N-terminus of the human ferritin heavy chain through a linker (SSG) and inserted into the MCS1 of the pACYCDuet-1 vector, as shown in Figure 3A. EDIII ferritin protein was expressed and purified according to the strategy used for EDIII protein purification. However, the molecular weight of the purified protein ${(}_{\sim }^{\sim }$ 17 kDa), as detected through the SDS-PAGE analysis, was not similar to the expected molecular weight (35 kDa) (Figure 3B). Western blot analysis of purified protein with anti-His antibody also confirmed that most proteins were expressed near the 17 kDa level, while a comparably low expression of the full construct was observed at the 34 kDa level (Figure 3C). To confirm whether the 17 kDa band was the EDIII protein cleaved from the full construct, the membrane was detected with an anti-DENV2 EDIII antibody, thus confirming that the 17 kDa band was derived from EDIII (Figure 3D). Considering these observations, it was confirmed that EDIII protein was separated from ferritin heavy chain protein through an internal mechanism. In our basic ferritin studies, we confirmed that the addition of a C-terminal 6 His tag could stabilize the full construct (Figure S1). Based on this phenomenon, a new EDIII ferritin construct was generated by inserting a C-terminal His tag (Figure 3E). Expression of the EDIII ferritin 6 His construct was confirmed by Western blotting, and the molecular weight of the detected band with the His antibody was similar to the theoretical molecular weight of the construct (35 kDa), while the cleaved EDIII protein was not detected (Figure 3F). Furthermore, SDS-PAGE was performed to determine whether invisible cleavages were formed; thus, only one major band was detected, and the molecular weight of this band was similar to that obtained by Western blotting (Figure 3G). Finally, the purity of purified EDIII and EDIII ferritin was analyzed by SDS-PAGE and proteins with exceptional purity and stability were observed (Figure 3H).

### 3.5. Confirmation of the EDIII Ferritin Nanoparticles

**Figure 4:**
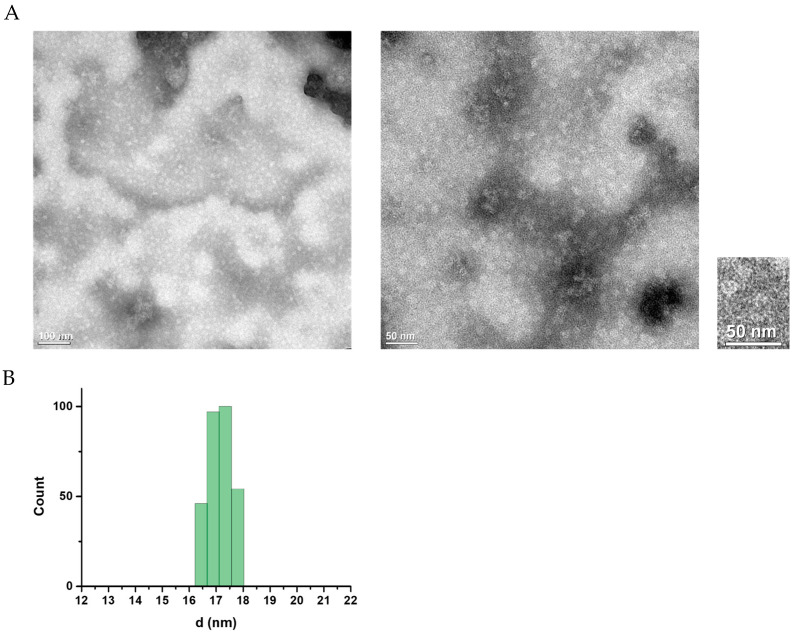
Characterization of purified EDIII ferritin nanoparticles. (**A**) White, round EDII ferritin nanoparticles were observed using a transmission electron microscope. (**B**) Size distribution patterns of EDIII ferritin nanoparticles were analyzed using DLS, and the average size of the particles was determined.

Particle characterization of EDIII ferritin was performed using TEM and DLS assays. White and spherical ferritin particles were detected by TEM analysis, and the particle size patterns were evenly distributed throughout the image (Figure 4A left panel). Furthermore, analysis of the particles with the highest magnification revealed the characteristic features of ferritin nanoparticles (Figure 4A, right panel). The DLS analysis revealed an average size distribution of 17.5 nm for the EDIII ferritin nanoparticles, indicating the unique size of purified ferritin nanoparticles (Figure 4B).

### 3.6. IgG ELISA and Virus-Neutralizing Activity

**Figure 5:**
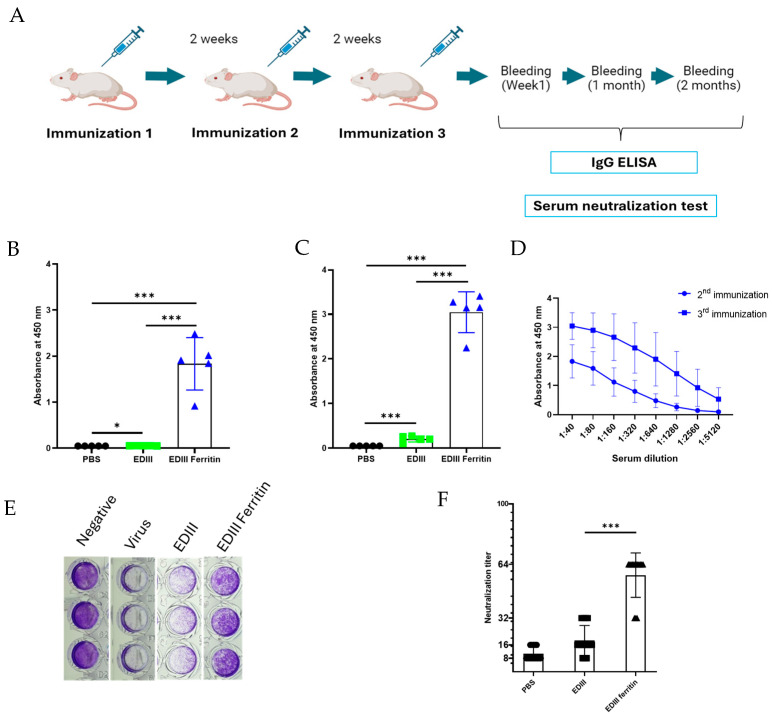
Mouse immunization and immune response analyses using IgG ELISA and virus neutralization assays. (**A**) Mice were immunized at the mentioned intervals, and blood was collected 1 week after the second immunization and at the mentioned intervals. (**B**) IgG ELISA was performed using serum obtained one week after the second immunization (serum dilution 1:100). (**C**) IgG ELISA was performed using serum obtained one week after the third immunization (serum dilution 1:100). (**D**) IgG ELISA results from the serum obtained 1 week after the second and third immunizations with EDIII ferritin were compared. (**E**) For the virus neutralization assay, DENV2 virus (3 × 10^3^ TCID50/mL) and diluted serum were mixed and inoculated into Vero cells, which were cultured in 96-well plates, and the cytopathic effect was analyzed by staining the plates with 0.05% crystal violet solution. (**F**) The neutralizing titer, which is the serum dilution that inhibited 90% of the viruses, was calculated and plotted. The significance difference in the immune responses was calculated using the Student’s *t*-test (*p* < 0.001 = ***, *p* < 0.05 = *).

The mouse immunization and bleeding schedules are shown in Figure 5A. Using the serum obtained one week after the second immunization and one week after the third immunization, EDIII-specific IgG ELISA was performed to confirm the specific immunity. After the second immunization, the average absorbance values of the PBS, EDIII, and EDIII ferritin-immunized groups were 0.049, 0.0528, and 1.8328, respectively. This confirmed that EDIII and EDIII ferritin induced significant specific IgG titers compared with the PBS control group, and EDIII ferritin induced significant IgG titers compared with the EDIII-only immunized group (Figure 5B). The ELISA results obtained after the third immunization showed average absorbance values of 0.05, 0.20, and 3.0, respectively, for the PBS, EDIII, and EDIII ferritin groups (Figure 5C). The average absorbance values of EDIII and EDIII ferritin were comparably higher than the absorbance values obtained from the second immunization, and both EDIII and EDIII ferritin induced significant IgG titers compared to the PBS group. Total IgG responses at both time points indicated that EDIII ferritin induced a significant IgG titer compared to the EDIII group. Furthermore, a comparison of IgG responses from the second and third immunizations of EDIII ferritin at each serum dilution showed that the third immunization produced significant IgG titers compared to the second immunization (Figure 5D).

The infected and cell-detached wells are represented in white, whereas the non-infected live cell-containing wells are represented in purple (Figure 5E). The maximum titer that reduced the CPE by 90% was expressed as the neutralizing titer. The average neutralizing activity of EDIII-immunized mouse serum was 1:16 and was distributed between 1:32 and 1:8 (Figure 5F). The EDIII ferritin-immunized mouse serum showed a significant neutralizing titer compared to EDIII only, with an average neutralizing titer of 1:64 (Figure 5F).

### 3.7. Longevity of Immune Responses Induced by EDIII Ferritin Nanoparticles

**Figure 6:**
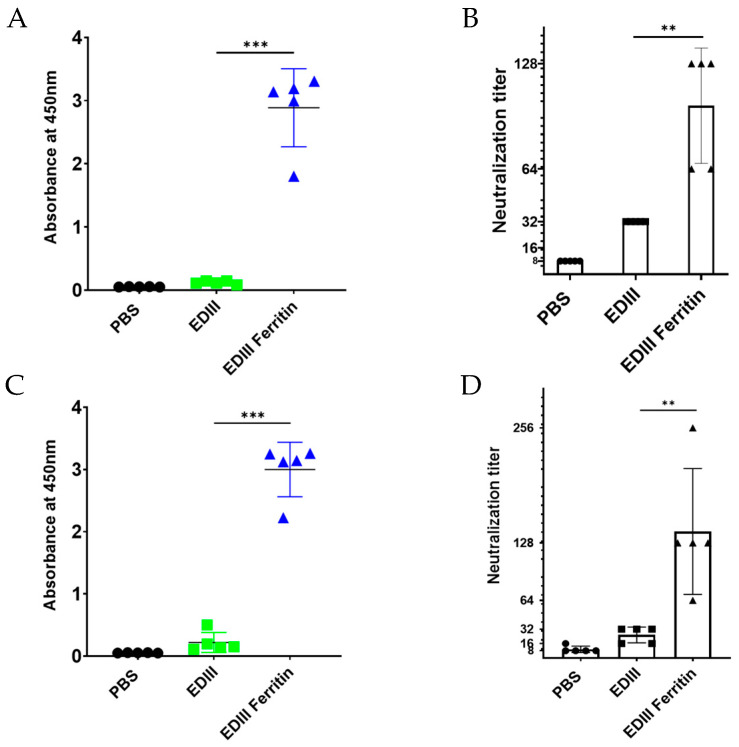
EDIII-specific IgG titers and DENV2 neutralizing titers 1 and 2 months after immunization. (**A**) EDIII-specific IgG titers were analyzed one month after the third immunization. (**B**) The virus-neutralizing titer, which is the serum dilution that inhibited 90% of the viruses, was analyzed a month after the third immunization. (**C**) EDIII-specific IgG titers were analyzed two months after the third immunization. (**D**) Virus-neutralizing titers were analyzed two months after the third immunization. The significant difference in the immune responses was calculated using a Student’s *t*-test (*p* < 0.001 = ***, *p* < 0.01 = **).

To confirm the durability of the generated immune response against DENV2, the IgG titer and virus-neutralizing activity were evaluated 1 and 2 months after immunization. One month after immunization, the IgG response of EDIII ferritin was significantly higher than that of EDIII, indicating average O.D. values of 0.11 and 2.88, respectively, for EDIII and EDIII ferritin (Figure 6A). Furthermore, analysis of the DENV2 virus-neutralizing effect of the same serum resulted in significant neutralization activity of the EDIII ferritin-immunized mouse serum. Two samples showed a neutralizing titer of 1:64, whereas three samples showed a neutralizing titer of 1:128 (Figure 6B). Moreover, analysis of the serum obtained 2 months after immunization resulted in a significant IgG response from the EDIII ferritin-immunized group. EDIII showed an average O.D. value of 0.21, while EDIII ferritin was 2.99 (Figure 6C), and the virus-neutralizing activity of the EDIII ferritin-immunized mouse serum obtained in month two was also significantly higher than that of the EDIII-immunized mouse serum. The neutralizing titer of EDIII ferritin was distributed between serum dilutions 1:256 and 1:64 (Figure 6D), resulting in 90% virus inhibition.

### 3.8. Obtaining Purified DENV3 EDIII Protein Using the Novel Strategy

**Figure 7:**
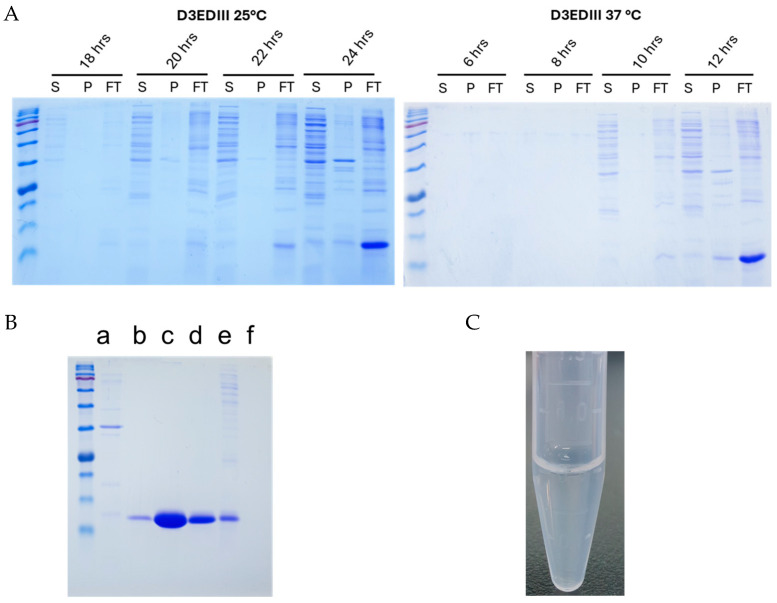
Purification of DENV3 EDIII using the modified method: (**A**) DENV3 EDIII-transformed *E. coli* was cultured in autoinduction media at two different temperatures (25 °C and 37 °C), and cells were harvested at different time points, followed by analyses of the expression and solubility of DENV3 EDIIII. (S—sonicated supernatant in PBS; P—freezing and thawing pellet; FT—freezing and thawing supernatant) (**B**) EDIII proteins were purified using the modified purification method, and samples from different purification steps were run on an SDS-polyacrylamide gel. (a—control BL21 *E. coli*; b—His affinity purified elute; c—concentrated EDIII protein using 10 K filter; d—aggregated or dimerized EDIII protein retained in 30 K filter; e—His-purified flow off; f—eluate from 10 K filter) (**C**) Buffer 2 was used to refolding and dialysis of DENV3 EDIII protein, and protein aggregates were eliminated.

The optimized protein expression and purification conditions were applied to purify the DENV3 EDIII protein to demonstrate the potential of these methods for the purification of other proteins. A DENV3 EDIII protein-expressing plasmid was generated using the same expression vector pACYCDuet1. First, the expression and solubility of EDIII protein were confirmed at different time points at 25 °C and 37 °C (Figure 7A). The expression of EDIII as a soluble protein or insoluble inclusion body was not detected after incubation at 25 °C for 22 h. However, a significant expression of solubilized proteins was observed 24 h after inoculation. At 37 °C, sufficient expression of the EDIII protein was achieved 12 h after inoculation. The purified, dialyzed, and concentrated proteins were evaluated using SDS-PAGE (Figure 7B), and plenty of purified proteins were obtained for DENV3 EDIII from the 10 K Amicon centrifugal filter units (Figure 7B). Moreover, the DENV3 EDIII protein, which was purified and refolded using buffer 2, did not induce any aggregation when stored at 4 °C, indicating the applicability of buffer 2 for future protein purification as an optimum refolding buffer (Figure 7C).

## 4. Discussion

In the case of protein expression in prokaryotic systems, a major problem is that target proteins accumulate in insoluble aggregates (inclusion bodies) without proper folding, resulting in biologically inactive proteins [25,26]. In this study, the optimization of critical factors to solubilize the inclusion bodies of DENV2 EDIII, obtain purified proteins, and eliminate the formation of post-purified protein aggregates was extensively discussed, and the particle characteristics and immunogenicity of EDIII ferritin nanoparticles were elucidated using in vivo studies.

Dengue viruses contain four serotypes with distinct genetic structures, which have a 65–75% similarity of the nucleotide sequence similarity, and all serotypes have the potential to cause dengue disease [27]. Infection with one serotype results in lifelong immunity against the respective serotype; however, it does not protect against other serotypes [28]. Thus, DENV disease enhancement can be caused by infection of other serotypes of DENV because of the condition known as antibody-dependent enhancement [29]. Therefore, the use of a complete envelope protein is not recommended in the development of subunit vaccines. Instead of the full envelope protein, the EDIII protein with major neutralizing antibody epitopes has been employed to reduce ADE [14]. Therefore, DENV2 EDIII protein was used as an antigen to develop a ferritin nanoparticle vaccine against DENV2. Self-assembling ferritin nanoparticles have been widely used in vaccine design. The human ferritin heavy chain has been extensively studied in the past decade and has been applied in various biomedical applications, such as vaccine development, drug delivery, and imaging [30,31,32,33]. Therefore, human ferritin can be used in human applications with enhanced biocompatibility. Here, we used the N-terminus of the human ferritin heavy chain to link the EDIII molecule because it has been shown that the N-terminal insertion of external molecules stabilizes ferritin nanoparticles compared to C-terminal insertion, as reported previously [34]. However, in this study, we found that EDIII and ferritin molecules could be partially or completely separated into two proteins without any external effects (Figure 3B). In our preliminary experiments on dengue EDIII protein conjugated with an insect ferritin heavy chain and a C-terminal 6 histidine tag, we confirmed the production of full-length EDIII ferritin protein (Figure S1). The same cloning strategy was applied to the human ferritin heavy chain in this study. Fortunately, this 6-histidine tag addition to the C-terminus of the EDIII human ferritin heavy chain construct could overcome the cleavage effect and stabilize EDIII ferritin (Figure 3F). However, the mechanism underlying this phenomenon remains unclear.

When considering the freezing and thawing method to recover insoluble proteins, it is a great alternative to methods applying high urea (6 M, 8 M) buffers. Previously, it was reported that the high urea content in the buffers could affect the tertiary structural changes and instability of the protein, leading to complications in chromatographic behavior [35]. The freezing–thawing method includes two types of buffer containing 1 M urea and 2 M urea, and it can cause a lower effect on the protein structure compared with the 6 M urea buffer. Acknowledging this, we suggested the 2 M urea freezing–thawing method for the solubilization of inclusion bodies of EDIII with exceptional purity (Figure 1E). EDIII proteins were obtained without impurities using the freezing–thawing method and affinity purification (Figure 2A). However, as shown in Figure 2B, many protein aggregates were observed after storage at 4 °C, which is common for other proteins as well as the EDIII protein. According to these observations, many *E. coli*-expressed proteins produced protein aggregates after purification through affinity purification, and these aggregates were not eliminated by simple dialysis in PBS. Therefore, the solubilization of inclusion bodies is not the only critical factor for purification, and the elimination of aggregation in purified proteins is essential. During protein expression, misfolded proteins tend to form aggregates, which can cause inclusion bodies to be solubilized and purified, leading to reaggregation. This reduces protein activity [36]. Therefore, protein stabilization methods should be developed by integrating the correct pI, pH, buffer composition, helper molecules, and the duration of the refolding process [37]. The pH values of the dialysis buffers were maintained at one unit above or below the pI values obtained for a specific protein. L-arginine is a prominent stabilizing agent that eliminates the misfolding of aggregation-prone proteins [38]. Moreover, glycerol has been identified as an aggregation-inhibiting protein that stabilizes the more compact state of its native structure [39]. Therefore, the dialysis system was modified with L-arginine and glycerol as the predominant protein-stabilizing agents, and an appropriate pH was maintained. Under optimal conditions, a stabilized EDIII protein was obtained (Figure 2D). Because protein subunit vaccines are delivered as proteins diluted in the same buffer, it is essential to consider the toxicity of buffers that solubilize proteins before immunizing mice. Thus, reduced toxicity can result in efficient immune responses to purified antigenic molecules without external effects. Our results revealed that the reduction of molar content of L-arginine and glycerol in the refolding buffer could also have a protein-stabilizing effect and especially lower cell toxicity compared with buffer 1, which contained high levels of L-arginine and glycerol. The employment of this buffer (buffer 2) in refolding EDIII ferritin and DENV3 EDIII proteins (Figure 7C) demonstrated the potential for the elimination of protein aggregates, similar to buffer 1. Therefore, this strategy can be applied to future protein production with high stability at a laboratory scale.

Immunization of mice with purified DENV2 EDIII ferritin nanoparticles induced a significant immune response against DENV compared to EDIII alone. To compare the immunostimulatory activity of the monomers and multimers in the ferritin cage, it is essential to use similar molar ratios rather than a similar amount (µg) of proteins because the volume acquired by one ferritin molecule is not similar to that of the EDIII molecule, delivering a diverse number of protein molecules. Therefore, molar-equivalent immunization has been used in multiple studies related to ferritin nanoparticle vaccines [30,40,41,42].

The potential immune stimulation of ferritin nanoparticles without using an adjuvant and preferences for adjuvant-free vaccines in the delivery of vaccines with low toxicity have been elucidated [41,43]. Based on these studies, mice were immunized without an adjuvant to elucidate the true effect of the purified protein on vaccination. With the results obtained by immunization with stabilized proteins, better IgG and neutralizing activity of EDIII ferritin were discovered (Figure 5), while the longevity of the immune response was also promising compared to EDIII only (Figure 6). Ferritin is a well-established biological structure composed of multiple subunits of ferritin heavy and light chains, or one form of ferritin. Conjugating an antigen with one ferritin subunit can produce multiple copies of antigens on the surface of the ferritin cage when assembled naturally. These antigen-displayed ferritin molecules mimic the structure of a virus and represent the antigen molecules efficiently to the B cells, inducing the humoral immune response and establishing multiple and prolonged binding with B cells compared to single antigenic molecules, followed by inducing a robust immune response [8,22]. However, it is essential to conduct a T-cell response analysis to confirm the activation of cellular immunity through this activation process. Furthermore, mouse challenge studies and cross-reactivity with other serotypes need to be analyzed to gain an overall understanding of the immune response.

This study provides valuable insights into laboratory-scale viral protein expression in *E. coli*, purification, and modifications for protein refolding, as well as a comparison of EDIII and EDIII ferritin immunogenicity. The principles behind this study will have a significant impact on the development of new vaccine strategies against dengue and other viruses.

## Data Availability

Represented data can be provided upon reasonable request by the corresponding author.

## Supplementary Materials

The following supporting information can be downloaded at: https://www.mdpi.com/1999-4915/17/1/129/s1, Figure S1: Comparison of EDIII insect ferritin heavy chain (iFHC), which contains a C-terminal 6 histidine tag and EDIII human ferritin heavy chain (hFHC), which does not contain a C-terminal 6 histidine tag.
