# Supplementary material for: Optimization of Conditions for Expression of Dengue Serotype 2 EDIII Protein in Escherichia coli and Immune Responses of Adjuvant-Free EDIII Ferritin Nanoparticles Against Dengue Virus in BALB/c Mice
Source: Viruses. 2025 Jan 17;17(1):129. doi: 10.3390/v17010129 (PMC12128699; doi:10.3390/v17010129)
Supplement: Supplementary file 1 [file viruses-17-00129-s001.zip › viruses-3360717-supplementary.pptx]

## Slide 1
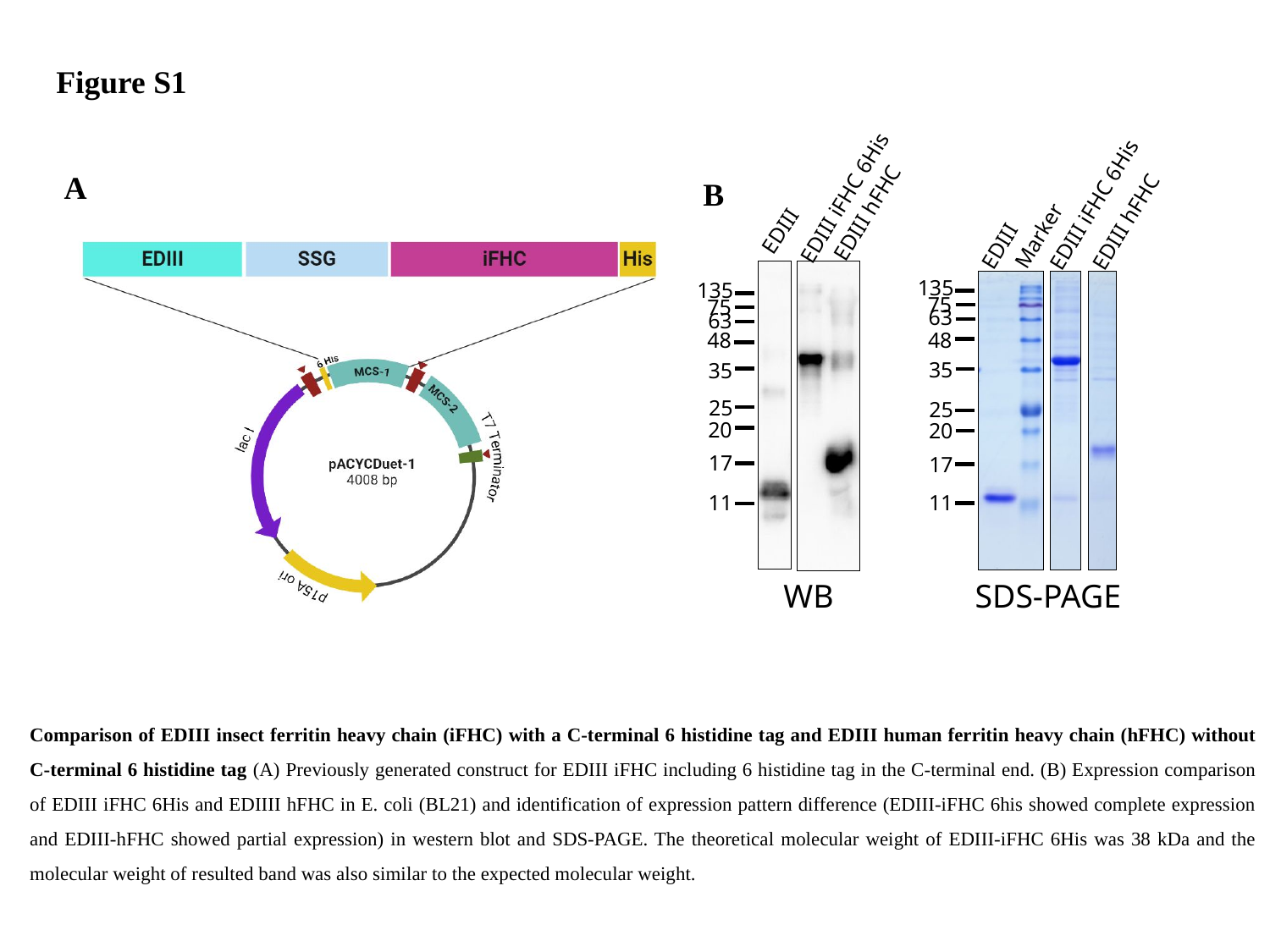

Figure S1
EDIII
135
75
63
48
35
25
20
17
11
EDIII iFHC 6His
EDIII hFHC
EDIII iFHC 6His
EDIII hFHC
EDIII
135
75
63
48
35
25
20
17
11
A
B
Marker
SDS-PAGE
WB
Comparison of EDIII insect ferritin heavy chain (iFHC) with a C-terminal 6 histidine tag and EDIII human ferritin heavy chain (hFHC) without C-terminal 6 histidine tag (A) Previously generated construct for EDIII iFHC including 6 histidine tag in the C-terminal end. (B) Expression comparison of EDIII iFHC 6His and EDIIII hFHC in E. coli (BL21) and identification of expression pattern difference (EDIII-iFHC 6his showed complete expression and EDIII-hFHC showed partial expression) in western blot and SDS-PAGE. The theoretical molecular weight of EDIII-iFHC 6His was 38 kDa and the molecular weight of resulted band was also similar to the expected molecular weight.
